# Professional regulation in the digital era: A qualitative case study of three professions in Ontario, Canada

**DOI:** 10.1371/journal.pone.0303192

**Published:** 2024-05-10

**Authors:** Kathleen Leslie, Sophia Myles, Abeer A. Alraja, Patrick Chiu, Catharine J. Schiller, Sioban Nelson, Tracey L. Adams

**Affiliations:** 1 Faculty of Health Disciplines, Athabasca University, Athabasca, Alberta, Canada; 2 School of Sociological and Anthropological Studies, University of Ottawa, Ottawa, Ontario, Canada; 3 Faculty of Nursing, University of Alberta, Edmonton, Alberta, Canada; 4 School of Nursing, University of Northern British Columbia, Prince George, British Columbia, Canada; 5 Faculty of Nursing, University of Toronto, Toronto, Ontario, Canada; 6 Department of Sociology, Western University, London, Ontario, Canada; University Hospital Cologne: Uniklinik Koln, GERMANY

## Abstract

Technology is transforming service delivery and practice in many regulated professions, altering required skills, scopes of practice, and the organization of professional work. Professional regulators face considerable pressure to facilitate technology-enabled work while adapting to digital changes in their practices and procedures. However, our understanding of how regulators are responding to technology-driven risks and the impact of technology on regulatory policy is limited. To examine the impact of technology and digitalization on regulation, we conducted an exploratory case study of the regulatory bodies for nursing, law, and social work in Ontario, Canada. Data were collected over two phases. First, we collected documents from the regulators’ websites and regulatory consortiums. Second, we conducted key informant interviews with two representatives from each regulator. Data were thematically analyzed to explore the impact of technological change on regulatory activities and policies and to compare how regulatory structure and field shape this impact. Five themes were identified in our analysis: balancing efficiency potential with risks of certain technological advances; the potential for improving regulation through data analytics; considering how to regulate a technologically competent workforce; recalibrating pandemic emergency measures involving technology; and contemplating the future of technology on regulatory policy and practice. Regulators face ongoing challenges with providing equity-based approaches to regulating virtual practice, ensuring practitioners are technologically competent, and leveraging regulatory data to inform decision-making. Policymakers and regulators across Canada and internationally should prioritize risk-balanced policies, guidelines, and practice standards to support professional practice in the digital era.

## Introduction

Technology is rapidly transforming service delivery across regulated professions, altering required skills, scopes of practice, and the organization of professional work [1–5]. Regulatory bodies that govern these professions are under immense pressure to adapt their practices and procedures to facilitate technology-enabled work, while also upholding their mandate to protect the public interest. This urgency has been magnified by the COVID-19 pandemic, which expedited the shift towards virtual practice [6–8] and the scrutiny of professionals’ social media behaviour [9, 10]. Regulators must evolve alongside technological advancements to mitigate emerging risks and ensure that new technologies do not negatively impact professional practice or services provided to the public.

Research exploring the impact of digitalization, artificial intelligence (AI), and other technological changes on professional work is emerging [11–15]. This literature has explored the interplay between traditional professional practice and digital innovation [16, 17], the organizational changes induced by digital development [13, 15, 18, 19], and the emerging digital competencies required by professionals [20, 21]. Our previous research has also identified challenges that accompany the expansion of virtual practice, including uncertainty about professional standards, breaches of privacy law and other legal requirements, and concerns about equity and professional ethics [6, 22, 23]. Furthermore, while AI technology has the potential to deliver more efficient and data-driven professional services, the associated risks are just beginning to be explored [24, 25]. Notably, the burgeoning use of AI raises issues around privacy, equity, and the integrity of professional practice, all of which have implications for professional regulators. Already there are cases where regulators have needed to respond to the inappropriate use of AI in professional practice [26]. Technology with the potential to profoundly affect professional practice calls for regulatory practices that are responsive to the changing sociotechnical landscape, including the associated risks, inequities, and uncertainties.

Despite emerging research on digitalization’s impact on professional work, our understanding of how regulatory bodies are responding to technology-driven risks and opportunities remains limited. Responses to these challenges have varied, with some regulators imposing a duty of technological competence [27], while others have refrained from setting formal tech-centric standards. Standards for virtual practice also vary significantly between regulators, even for the same profession [6]. In addition to how regulators are working to support registrants in the digital era, regulators may be adopting technology to improve their own regulatory practices. To varying extents, professional regulators are digitizing processes related to registration, complaints management, continuing competence, and discipline. However, the way that professional regulators may be using technology to enhance their own work has received little research attention.

This research aims to address these research gaps by examining how regulatory practices and policies are evolving in response to digitalization, and how regulators can support professionals through significant workplace changes while upholding the public interest. Building on our team’s recent work, including a knowledge synthesis of existing academic and grey literature [22, 23] and a review of regulatory guidance around virtual care [6], this study provides foundational insights into the challenges and opportunities of regulating in a digital era. Understanding these challenges and opportunities can inform professional regulators across various sectors and jurisdictions and guide the development of risk-appropriate policies, guidelines, and standards for modern professional regulation.

## Objectives

We aimed to examine the impact of technology and digitalization on three regulatory bodies in Ontario, Canada through a qualitative comparative case study approach. Our specific objectives were to:

1. Explore approaches and policies that these regulators have enacted to address the challenges and opportunities presented by the digital era;
2. Describe the effects of technological change on regulatory activities at these regulatory bodies; and
3. Compare the regulatory responses to technological change across these regulators.

## Methods

Our study was guided by Yin’s [28] structured approach to exploratory case study research, which is well suited to understanding new or less studied areas where the researcher has little or no control over the phenomenon of interest. Case study designs in professional regulatory research can produce comprehensive analyses for examining regulatory reforms and innovations [29]. In this study, we compared how three professional regulators in Ontario, Canada approached regulating in the public interest in the digital era.

### Participants

Participating regulators were the Ontario College of Social Workers and Social Service Workers (OCSW), the Law Society of Ontario (LSO), and the College of Nurses of Ontario (CNO). As an exploratory case study, we sought a variety of professional sectors; law, nursing, and social work offered a diversity of regulatory and professional contexts. These three regulators are each under separate legislative frameworks but share the common context of a single jurisdiction of Ontario. Further, the nature of the professional work being regulated is different, and we expected that technology may be impacting these different work contexts in unique ways. In this way, we could distinguish between issues specific to each regulatory body and cross-cutting themes present across the professional, legislative, and workforce factors that vary between them. Our selection of these three regulatory bodies was thus aimed at achieving what Flyvbjerg [30] called maximum variation in case study research and is appropriate for exploratory work that can be built upon by examining other professions in more jurisdictions in future projects. A brief description of these regulatory bodies is provided in Table 1.

**Table 1:** Professional regulators in Ontario, Canada included in the study.

Regulatory Body	Abbreviation used for Attribution	Number of Representatives Interviewed	Categories of Professional(s) Regulated	Legislative Framework
Ontario College of Social Workers and Social Service Workers	OCSW	Two	Social Workers Social Service Workers	*Social Work and Social Service Work Act*, *1998*
Law Society of Ontario	LSO	Two	Lawyers Paralegals	*Law Society Act*, *1990*
College of Nurses of Ontario	CNO	Two	Registered Nurses Registered Practical Nurses Nurse Practitioners	*Regulated Health Professions Act*, *1991* *Nursing Act*, *1991*

### Data collection

We conducted data collection over two phases. The first phase involved gathering publicly available documents relevant to our research objectives and the second phase involved semi-structured interviews. We started with documents to gain a foundational understanding of how technology intersected with regulatory policy and practice for these three professions, and our interviews provided further insight and contextualization to deepen our understanding.

In the first phase, we identified relevant documents by searching regulators’ websites. We conducted our initial searches between May 2022 and September 2022, reviewing documents back to 2015. The date of 2015 was chosen to capture pre-COVID responses while being feasible given our research funding timeline. We also anticipated limited applicability and availability of older regulatory documents given the rapid change of technology in the past decade. The search was updated in January 2023.

We searched each regulator’s website for keywords based on concepts that fit with our research objectives (e.g., digital, tele*, virtual, tech*) and then specific documents were reviewed in full, including minutes from council meetings, regulatory publications for registrants and the public such as newsletters, and strategic plans. We reviewed each retrieved document for references to other regulatory documents, webpages, or resources that were directly relevant to the impact of technology on the regulator’s policies or practices. In this iterative way, we retrieved regulatory updates and news releases; frequently-asked-questions; policies and policy briefs; council briefing reports and minutes; and practice statements, standards, directives, guidance, and guidelines. We identified 34 documents (nine from the CNO, 15 from the LSO, and ten from the OCSW; see S1 File) that met our inclusion criteria of relating to our research objectives and being published between 2015 and 2022. Document information was organized in a spreadsheet and copies of all included documents were saved as PDFs to keep a record in case websites changed during analysis. These documents allowed us to identify pivotal milestones and policies related to regulatory responses to technology that were used to inform the development of our interview guide. All documents were publicly available.

In the second phase, we emailed the executive director or registrar from each of the three regulators to explain the study and request an interview with individuals familiar with the regulator’s response to technology and relevant policies and practices. Each executive director agreed to an interview and designated two individuals (total n = 6) from their regulatory body to participate and with whom the research team communicated directly by email. Our sampling strategy was purposive and required study participants to have knowledge about the impact of technology on regulatory practice and policy at their organization, appropriate for our case study research design.

We developed a semi-structured interview guide and shared this with our participants prior to the interview (see S2 File). The semi-structured interview guide was developed collaboratively by the research team based on our previous research in the field, our recent scoping review on a similar topic, and our preliminary analysis of the Phase 1 documents. The first author (KL, she/her) is an early career researcher with experience conducting qualitative interviews in regulatory research who was supported by senior career qualitative researchers (TLA, SN). Interviews were conducted between 1 January 2023 and 31 March 2023 by the first author (KL), and at least one other research team member attended and took detailed notes (TLA, AA, SM). At the beginning of each interview, the first author (KL) provided participants with background information and reiterated the study purpose and objectives. Interviews were conducted by videoconference software (Microsoft Teams) and lasted approximately 60 minutes. Each interview was recorded and transcribed verbatim.

### Data analysis

Following data collection, we analyzed all data (documents, interview transcripts, and field notes) using thematic data analysis [31, 32]. In this flexible but rigourous approach to data analysis, the researchers play a key role in knowledge production through prolonged engagement with the data [32]. Three research team members (KL, AA, SM) independently read all documents, transcripts, and field notes to develop a coding framework, using the categories and concepts from the documents and semi-structured interview guide as a basis. Two research team members (KL, PC) with experience in qualitative data analysis and regulatory research then coded all transcripts independently by hand using colour-coded highlighting. KL and PC collaborated throughout regular discussions to develop themes. Extracts from transcripts were brought together for each theme as it was developed, and information from the documents was used to augment and triangulate these findings. As results were developed for each regulator, we compared and contrasted findings across the cases to identify themes and develop rich descriptions. Finally, we returned to the original transcripts and audio recordings to ensure the identified themes and selected quotes accurately reflected the data. We also identified documentary data from Phase 1 that could be included to support the findings and add context, depth, and breadth to the interview data. Findings were regularly reviewed and refined with the research team throughout the analysis.

### Trustworthiness

To ensure the credibility of our findings, we used a multifaceted approach. Data collection and analysis were carried out in a systematic way, including extensive engagement with the transcripts and generating a careful audit trail. Using multiple data sources provides enhanced validity with our findings supported by more than one source of evidence [28, 33]. We met regularly as a research team to discuss and verify our analysis; we included thick descriptions of our findings and verbatim quotes to support each theme. The diversity of our team, which includes research and clinical backgrounds in professional regulation, political science, nursing, law, and sociology, provided a range of analytical perspectives to aid in interpreting results. We followed the Consolidated Criteria for Reporting Qualitative Research (COREQ) [34] to guide the transparent reporting of our methods and findings (see S3 File).

### Ethical considerations

The Athabasca University Research Ethics Board approved the study (File #24794). Each regulatory body contacted agreed to participate and informed written consent was obtained from all interview participants. No one withdrew their participation and only participants and research team members were present during the interviews. The study posed minimal risk to participants as they provided information in their professional capacity and no personal information was collected. To protect participant confidentiality, quotes in the findings are attributed to the regulatory bodies and the professions they represent without using names or position titles. As each regulator represents a single case, we have not differentiated between participants representing the same regulator.

While some research team members are members of the profession of participating regulators, they do not have established relationships with interviewees (prior to study commencement or since study completion), and interviewees were aware of the principal investigator’s (KL) background as a registered nurse and lawyer.

## Results

We identified five themes in our analysis of the interview and document data: 1) balancing efficiency potential with risks of certain technological advances; 2) the potential for improving regulation through data analytics; 3) considering how to regulate a technologically competent workforce; 4) recalibrating pandemic emergency measures involving technology; and 5) contemplating the future of technology on regulatory policy and practice. Descriptions of these themes, along with illustrative regulatory responses and quotes, are provided in Table 2. We also review these themes in detail below, providing context from regulatory documents and quotes from interview transcripts. Quotations have been very lightly edited for grammar.

**Table 2:** Descriptions of themes with examples of regulatory responses and illustrative quotes.

Theme	Description	Examples of Regulatory Responses and Illustrative Quotes		
		Law Society of Ontario (LSO)	Ontario College of Social Workers and Social Service Workers (OCSW)	College of Nurses of Ontario (CNO)
**1. Balancing efficiency potential with risks of technological advances**	Evaluating the trade-offs between the increased efficiency offered by new technologies and the potential risks they pose. Emphasis on maintaining a balance between human interaction and automated processes.	Moved to online applications and certain automated processes. “ensuring that we’re approaching things from a risk-based perspective is very important” (LSO)	Considering moving to digital certificates; however, concerns remain around fraud and technological tampering. “how do we ensure that it’s not fraudulent?” (OCSW).	Exploring automation for certain low-risk tasks to improve response times and using technology to increase engagement with regulatory documents.
**2. Potential for improving regulation through data analytics**	Leveraging data to inform decision-making and enhance regulatory outcomes. Cautious but optimistic towards data analytics to support decision-making.	Developing new data-informed competence frameworks. “We always use data to…assess risk and try to get ahead of things” (LSO).	Transitioning to a new data system that will enable better data analysis. “we have the questions we want to ask” (OCSW).	Implementing an Insights Engine for real-time data analytics, though still in early stages. “[Insights Engine] will make us more efficient” (CNO).
**3. Regulating a technologically competent workforce**	Considering the appropriate level of technological understanding and ability regulators should expect from their registrants.	Adopted a technological competence requirement, with tailored guidance for different practice areas. Also offers guidance through a Technology Resource Centre.	Integrates technological competence into broader standards, avoiding overly specific guidelines. “Technology really doesn’t warrant its own standalone standard” (OCSW).	Considers standards broad enough to cover technological competence, with focus on supporting tech-enabled practice.
**4. Recalibrating pandemic emergency measures involving technology**	Adjusting regulatory practices in response to the pandemic’s push towards virtual operations and assessing what should be retained or revised, with an emphasis on equity considerations.	Experienced an exam cheating scandal and no longer doing online testing. Aiming for a hybrid approach to other regulatory functions post-pandemic. “The trick is to determine when [human engagement] is needed” (LSO).	Exploring how digitization might impact future entry-to-practice exams that are under consideration.	Did not adopt remote proctoring during the pandemic. Considering the impact of simulated clinical experiences on graduate competence.
**5. Future of technology in regulation**	Exploring how emerging technologies like AI will affect professional practice and regulatory policies.	Interested in how virtual proceedings and AI affect legal outcomes. Supporting innovative legal services through the Access to Innovation program’s regulatory sandbox.	Seeks to be more forward-thinking and collaborative with other regulators on technology use. “There’s no reason the regulator can’t be the cutting edge in some of this” (OCSW).	Interested in AI’s impact on the therapeutic relationship and considering the role of collaborative data sharing.

### Theme 1: Balancing efficiency potential with risks of technological advances

Participants recognized that technological advances may enable regulators to work more efficiently; however, they believed the risks associated with new technologies necessitated a cautious approach. This caution meant that many changes were incorporated slowly, and participants emphasized that even seemingly simple changes, such as online registration processes, were being approached carefully. A participant from the LSO noted that “historically, we’ve been very paper-based, so people would fill out paper applications and they would mail those into us” but the pandemic “accelerated changes that were being considered prior to that, and certainly desired prior to that, and so the application process has moved online.” Similarly, a participant from OCSW described their desire to move away from printing out physical certificates for all registrants, noting “we’re hoping we can transition out of it because it’s a very manual, expensive process that we do across the board for everyone.” However, the potential efficiency gain of digital certificates also came with risk, with the participant noting “then technology comes into play, because how do we ensure that it’s not fraudulent? … There’s always the ability to tamper with it” (OCSW).

Participants also discussed the importance of carefully considering the contexts in which technology should be used to facilitate regulatory operations. Participants from all three regulatory bodies discussed opportunities to automate tasks to free up staff and to improve their response times to registrants, especially for low-risk activities. For example, inquiries related to registration renewals are often straightforward and may not require human interaction. Participants from the CNO and OCSW discussed how technology can create opportunities for registrants to better engage with regulatory bodies and increase the accessibility of standards and guidelines at the point of care.

At the same time, all participants emphasized the need to consider the full context of each situation to assess the appropriate use of technology. One commonality between regulators was the recognition that not everything should be digitalized because regulation also involves a relational component. This requirement for human interaction is especially relevant when dealing with registrants and the public in higher-stakes situations (e.g., investigations and discipline processes). A participant from OCSW noted that technological interactions were appropriate for the “benign stuff” such as registration renewals, but that “there is a balance and if a regulator wants to be sort of relevant, they have to ensure that they are meeting that sort of human need.” A participant from the LSO responded similarly:

*I think*, *probably the answer is that where processes that don’t really require a human touch*, *so for instance*, *handing an application in–we can eliminate the human touch there and move those types of things increasingly online*. *But I think maintaining those sorts of areas where having someone to talk to where you’re having an ethical issue*, *having a CPD program that you can attend that speaks specifically to your practice area and there are other colleagues there as well*, *those types of things*. *I think*, *that’s probably–to my mind at least–where the balance is*. *You beef up those areas where [human interaction] is important*, *and you try to gain those efficiencies in areas where perhaps it’s not so important that you do something in person*

(*LSO*)

Participants’ responses highlighted the value regulators place on using a risk-based approach to carefully assess the suitability and feasibility of integrating technology into regulatory processes: “I think increasingly, as we try to move into new areas with technology, that ensuring that we’re approaching things from a risk-based perspective is very important” (LSO).

The potential of technology to improve efficiency was valued by participants who noted the complexity and resource-intensity of regulatory processes were increasing; however, the risk-adverse nature of regulation meant currently these gains have not yet been fully realized as participants focused more on the potential efficiency gains, should risks be appropriately managed.

### Theme 2: The potential for improving regulation through data analytics

In addition to potential efficiency gains, participants highlighted where data could be leveraged to guide decision-making and potentially improve regulatory processes and outcomes. Similar to the cautious approach regulators were taking to the incorporation of new technologies more generally, the discussion of data analytics focused primarily on potential, rather than realized, benefits.

Participants from the CNO discussed the development of a new Insights Engine [35] that focused on data analytics but was in the very early stages of being used. This new system is meant to provide “more real-time data” that would allow the regulator to “specifically tag pieces of information…where they are coming from, from whom, and that informs how we also develop resources for members” (CNO). A CNO participant noted that the regulator had “lots and lots of data inputs” but these were “not accessible in a way that is as meaningful as we would like them to be in order to drive…our projects or the things that we need to improve or change.” The CNO participant described this as important not only to inform decision-making but to “prioritize our projects, where we kind of put our resources and our people, kind of focus our work so I think that will make us more efficient.”

A participant from the OCSW also noted a change in their current data systems that could, in the future, support data analytics for decision-making:

*We’re changing over from our current legacy software into a new CRM [customer relationship management system] this year…we are hoping [the new CRM] will be able to mine the data in that way and be able to identify trends*. *So even when we’re looking at complaints*, *registrants*, *investigations or even something simple like [continuing competence program] completion rates*. *We have a pretty high completion rate*, *but let’s say we do have a situation where we have lower completion rates*, *we can look to see if there are certain regions that have individuals that are not participating as much or things like that*. *Currently*, *we can’t do that as simply*, *but in a future state*, *we’d want to*. *So*, *we have the questions we want to ask*.

(*OCSW*)

In addition to regulators’ plans to use data analytics to improve regulation, we also heard that regulators were using existing data to identify practitioners at risk. As a participant from the LSO noted, “I think we always use data that’s available to us to sort of assess risk and try to get ahead of things and understand where problems exist”. For example, existing data have “clearly shown for many years that the sole practitioners are at the highest risk of being the subject of complaints” and “the causes of this are usually skills-related, not knowledge of the law” (LSO). Using this data, the regulator developed a new competence framework to support the implementation of a practice essentials course to support sole practitioners; at the time of our data collection, this was almost ready to be rolled out.

### Theme 3: Considering how to regulate a technologically competent workforce

Beyond considering how to incorporate technology to improve regulatory operations, participants also discussed how to support and regulate their registrants in an increasingly digitalized world. Participants from all three regulatory bodies discussed the factors that have influenced their regulatory approaches as technology changes how their registrants practiced. Participants discussed the importance of assessing the most appropriate level of regulation to balance their mandate of protecting the public while ensuring that they do not create barriers in practice. This is sometimes termed right touch or risk-based regulation.

One key consideration was the legislative framework under which regulatory bodies operate and how this impacted regulators’ ability to respond nimbly. A participant from the LSO described their broad bylaw-making power under the *Law Society Act* and the way it allowed the regulator “quite a bit of flexibility…[to] be fairly nimble when we need to be…basically to allow access to justice to continue when no one could meet in person” during the pandemic. For example, the LSO facilitated emergency Orders in Council to allow virtual execution and witnessing of wills [36] provided legislative interpretation regarding virtual client identification [37], and guided registrants about requirements for virtual notarizing and commissioning [38].

Other factors influencing regulators’ decision-making included their existing strategies for modernizing standards and the influence of collaborative work between regulators. Participants from the CNO highlighted pre-pandemic work that was done in collaboration with other regulators to develop guidelines and competencies for compassion in the digital healthcare age. This work informed their ongoing reforms for technology-enabled practice and virtual care as the regulator worked to modernize practice standards [39].

Similarly, a participant from the LSO spoke about the influence of the Federation of Law Societies of Canada on discussions around technological competence. In 2019, this Federation added commentary to the duty of competency in its Model Code of Professional Conduct; this commentary required lawyers to have an understanding of and ability to use technology relevant to the nature and area of the lawyer’s practice and responsibility [40]. The commentary further required that lawyers understand the risks and benefits associated with relevant technology. Different legal regulators across Canada began to adopt this duty of technological competence, and the LSO adopted it in 2022. As one LSO participant described:

*In general*, *we try and stay fairly close to [the model code]*, *certainly with the basics*, *because*, *you know*, *that’s what a national model code is supposed to do*. *And in this one*, *we were actually slow to adopt it because we had a technological taskforce…And then we also had a competence taskforce—the competence taskforce was intended to look at if our competence standards and programs were suitable for the next 20 years…the tech competence requirement was part of that report…So that was the impetus for this particular change to our rules*.

(*LSO*)

An important element of this commentary to the competence rule is that legal professionals must understand technology relevant to their area of practice. One participant from the LSO discussed the importance of this approach given the nuances in different law practice areas. This participant noted that the regulator was not assessing technological competence because “depending on practice area, it’s often not entirely clear how we would assess it anyway” since some areas of legal practice required uses of very specific technologies (e.g., the Ontario Teranet system to transfer title in real estate law) “whereas there are other areas of law where, in theory at least, you could probably still get by with a typewriter and dictaphone” (LSO).

In contrast to the LSO’s specific inclusion of technology in its commentary to the duty of competence, the OCSW and CNO described how their current standards were broad enough to generally cover technological competence. The Council of the OCSW specifically decided not to adopt the model technology guidelines for social work published by the Association of Social Work Boards in 2015 [41], opting “instead to produce resources to help support members in their practice and to apply those standards to technology or any other issue” (OCSW). One participant from the OCSW described how this decision was made:

*I think one of the convincing reasons for that was the current edition of our standards of practice refers to diskettes*, *which is such a great example of why you don’t want standards of practice to be too specific*, *because they’ll be out of date before the ink dries*, *I guess*. *And so I think that has been the approach that our college has taken—that technology competence and technology really doesn’t warrant its own standalone standard because it applies to other standards such as record keeping boundaries*, *competence and integrity*, *and confidentiality*.

(*OCSW*)

Despite differences in approaches to technological competence, participants from all three regulatory bodies spoke about their role in supporting their registrants’ professional practice by developing guidance around technology and virtual practice. In deciding what practice resources were needed, regulators responded based on registrant inquiries and the level of perceived risk. For example, a participant from the LSO discussed how they use inquiries from their practice management helpline to inform their continuing professional development offerings and regulatory resources.

Participants from the OCSW discussed how they encourage registrants to identify learning needs associated with technology when completing their continuing professional development program. Our review of OCSW newsletters showed ongoing encouragement of members to review resources on topics such as using communication technology in practice, selecting online platforms for virtual services, and practice standard considerations for navigating COVID-19 [42–46]. Participants from the OCSW also highlighted measures they took to ensure social workers from other Canadian jurisdictions were registered with OCSW before providing virtual services to clients in Ontario. This enabled the regulator to address issues related to professional conduct since “we can’t minimize the risks involved in electronic practice—they’re huge…so it is probably extra important that those people be registered in Ontario” (OCSW).

In developing practice support and guidance, participants discussed whether they had a role in providing guidance on specific technologies. There were differences in perspectives between regulators. For example, participants from the CNO discussed that it was not within their mandate to endorse or provide guidance on specific technologies:

*Questions about which [technology platform] I should be using—we don’t regulate that*, *right*? *We don’t say one thing is better than the other*. *We don’t endorse certain products*. *So that also becomes a challenge as well*, *what do we tell them in order to assess what is appropriate*? *It goes back to your accountabilities*. *Does it meet those standards that you’re obligated to follow in order to assess whether this application or technology is appropriate and is safe for you to use*?

(*CNO*)

Participants from the LSO spoke about their technology resource centre [47] that provided guidance on selecting technology for practice, technology use, data protection, cybersecurity, cloud computing, and working remotely. While this was a departure from the level of guidance provided historically, this participant felt it was necessary to guide legal professionals on what was required to meet the standard of professional competence.

*We’ve also tried to sort of help people along*, *in terms of what was created at the Technology Resource Centre…that was sort of a bit of a departure for us*, *because we got into really looking at specific products and…even suggesting to people that these are products that you can use in certain areas to help facilitate your practice*. *And traditionally*, *that hadn’t been our approach*. *Our approach was much more of satisfy yourself—go out there and see what’s out there kind of approach*. *So*, *we’ve tried to help nudge people in the right direction and give them a better idea of what we mean by technological competence*. *But that has certainly been a challenge*.

(*LSO*)

Thus, while all three regulators operate within the same province, their approach to regulating a technologically competent workforce differed, creating differences in standards and resources provided to registrants regarding technology.

### Theme 4: Recalibrating pandemic emergency measures involving technology

In many ways, the COVID-19 pandemic accelerated the use of virtual technologies and impacted various aspects of regulatory operations, including entry-to-practice, registration, continuing competence, and complaints and discipline proceedings. Different perspectives emerged about whether specific regulatory functions should remain virtual or return to pre-pandemic in-person norms. A recurring theme was the need to strike a balance between both modalities.

*I think through all of this*, *whether it’s hearings or discipline or entry to practice*, *I think there’s increasingly this feeling that… some form of hybrid is probably where the sweet spot is*, *and I think we need to figure out where that is*, *and figure out what’s going to stay online*, *what’s going to stay remote and what should be in-person*. *And I suspect it’ll take some time to really figure that out*.

(*LSO*)

Since a core function of regulatory bodies is to ensure that applicants meet the required entry-to-practice competencies, regulators found themselves grappling with the consequences of changes to the delivery of education and practicums during the pandemic. Participants from the CNO discussed how the lack of evidence on the outcomes of simulated clinical experiences created challenges for ensuring graduates were prepared competently:

*Is there a break point in terms of safety*? *And there is research out there [but] none of it is conclusive*. *There certainly are some jurisdictions that have gone the route of saying*, *you know*, *50% of your clinical can be simulation*. *We haven’t designated a percentage yet*.

(*CNO*)

Participants from the LSO also experienced challenges as education, placements and articling shifted online. In some cases, applicants were called to the bar after completing their legal education and training remotely. While these changes were necessary due to the pandemic, participants discussed the need to re-assess to determine the best approach going forward.

The pandemic also meant that some regulators shifted to online entry-to-practice exams, but this raised risks related to the security of exams and the appropriateness of the testing environment. The CNO did not adopt remote proctoring and participants noted they “weren’t comfortable at the time with the kind of research that’s out there in terms of security and the whole process around remote proctoring. And we are well aware that there are other regulators—both nursing and outside of nursing—that are doing it. So it’s something we’re exploring” (CNO). The LSO was one of the regulators that adopted remote proctoring and experienced an “exam cheating scandal…where we received information and materials to suggest that there had been cheating involved in some of our licensing examinations…And we’re now no longer doing testing online.” While the OCSW does not currently require an entry-to-practice exam, participants spoke about their interest in exploring how digitalization might impact future exam requirements that were being considered.

In considering the use of technology to increase efficiencies during the licensure process, one participant from the LSO identified the need to attend to equity considerations:

*I think the other place where you really see that tension playing out is in the equity space … for*, *particularly*, *students who are going through the licensing process*. *If you are in one of these marginalized groups*, *you can … face more barriers*, *you can become more anxious about dealing with what feels like the behemoth*, *the impersonal behemoth*, *of the Law Society*…*So that’s a tension and because efficiency is highly valued*… *I think*, *how we are trying to deal with that is to have a personal engagement as needed*. *The trick is to determine when it’s needed*. *I don’t think it’s hard to find a sympathetic ear actually at the Law Society*, *but to know when that intervention is required—that’s*, *I think*, *the real challenge*.

(*LSO*)

In addition to changes to entry-to-practice and registration/licensure, regulators’ investigation, discipline, and conduct processes were significantly disrupted by the pandemic as many of these shifted from in-person to virtual formats. This shift to virtual formats provided some advantages that participants described, such as “levelling the playing field” (CNO) for those who lived far from the regulatory body and potentially easing anxiety to facilitate more open conversations.

Participants from the CNO and LSO highlighted the potential negative consequences of virtual hearings such as increased ‘Zoom fatigue’, perceptions of informality, and barriers for individuals who live in areas with unreliable internet access. A participant from the CNO mentioned concerns about whether virtual hearings would have the same level of seriousness as an in-person meeting. These concerns were shared by those at the LSO:

*When you walk into a courtroom*, *and you see that the judge sitting up there and there’s a sense of formality to it*, *versus getting on a Zoom call with a bunch of heads from shoulders up*. *That may not feel like you’re getting the same hearing that you might have in person*. *So*, *I think there’s definitely a balance there*.

(*LSO*)

In general, regulators seemed to be striving for a balanced approach to post-pandemic recalibrations. Advantages, disadvantages, and risks associated with in-person and virtual modalities are becoming clearer and are helping inform this recalibration process.

### Theme 5: Contemplating the future of technology in regulatory policy and practice

Participants described their curiosity in wanting to learn more about the impact on public protection as technologies like AI continue to evolve and become more commonplace. Participants described specific areas they were watching to learn more about how technology was impacting professional practice, such as how the therapeutic relationship might “look different when practicing in a more technological environment” (CNO). A participant from the LSO was interested in exploring “whether virtual proceedings have impacted incarceration rates, and release versus bail, or other outcomes” and whether digital products such as online “will kits” help people in the way they are intended.

Participants highlighted opportunities for increased collaboration between regulators to streamline and harmonize processes and to share data to support evidence-based regulatory policy across professions. For example, one participant from the CNO said “I think there’s always been that collaboration amongst different regulators, but having data, I think, provides this objective piece as well, which I think will be helpful for sure.” A participant from the OCSW described the importance of working together given the similarities in regulatory functions and services, especially across health profession regulators:

*In essence*, *even though we’re regulating different professions*, *we’re all regulating*. *So*, *when you’re thinking of technology and the use of social media and some of the expectations we have from our professionals*, *specifically health*, *it would be good if those regulatory bodies could come together and say*, *you know*, *here’s the spectrum of our expectations*. *Here’s how we’ve been monitoring this spectrum*. *Let’s talk about it as a unit…We may be regulating different professionals*, *but we’re all running organizations*. *The operations of each of these organizations are extremely similar and overlap*.

(*OCSW*)

A participant from OCSW expressed a desire for regulation to become more forward-thinking around technology: “Often regulators are sort of the last ones around the corner with a lot of the updates……there’s no reason the regulator can’t be the cutting edge in some of this.” One more innovative approach to staying on top of technological changes was the LSO’s Access to Innovation program which created a regulatory sandbox for innovative technological legal services [48]. While, at the time of our interview, this sandbox did not yet have evaluation data available, this way of providing a safe space to support innovative technology is meant to provide insight into new risks and opportunities related to technology.

## Discussion

Technology can potentially revolutionize professional work and the regulation of that work. However, technological change has simultaneously created tensions around the application of AI, data analytics, technological competence, social media use, and other dimensions of professional practice and regulation [49]. The results of our study have underscored the wide-ranging implications of technology on regulatory practices, with changes to entry-to-practice and registration/licensing, practice standards and guidance, practitioner support, continuing professional development, and disciplinary practices and procedures. Regulators are grappling with how to respond to technological changes in the practice of the professionals they regulate, while simultaneously experiencing technologically mediated changes to internal processes.

### Concerns about risk may slow progress

Our findings illustrate that regulators are in the early stages of exploring the opportunities and risks that technology has on daily operations. Although regulators from different sectors have adopted technology at varied paces, the literature suggests that many have begun exploring the potential value of technology, including AI, in supporting regulatory operations and decision-making [50–55]. Regulation is becoming increasingly complex and resource-intensive, and our findings suggest that participants are hopeful that integrating technology into regulatory operations will provide a means to enhance efficiency. While participants noted that there are areas where technology can be integrated more easily, they also highlighted the importance of carefully studying the impacts of technology on functions that carry a greater risk of producing undesirable outcomes. This finding aligns with research by van der Gaag et al. that found regulators were concerned that AI could introduce errors and biases, jeopardize public trust, and perpetuate systemic inequities [56]. Our participants noted a need to take a risk-based approach to the advantages and disadvantages of adopting technology to support decision-making. While risk aversion may be necessary for higher-stakes regulatory processes, it may slow progress in adopting technological enhancements that could drive needed efficiency gains in increasingly resource-constrained environments.

### Ensuring an equity-based approach to virtual practice and processes

The rapid expansion of virtual services influenced our participants’ responses to regulating technology use among professionals. In the context of the health and social care sectors, the pandemic accelerated the proliferation of virtual care options. In the context of law, evidence from before the pandemic suggests that, increasingly, lawyers are operating virtual legal offices rather than those of traditional brick-and-mortar [57]. Technology use in practice raises important questions about privacy, confidentiality, boundaries, informed consent, continuity of services, and new complexities for addressing malpractice and complaints [6, 57–59]. While our participants discussed that technology does not necessarily require the development of unique standards of practice, this is an area that regulators will be required to re-evaluate as new risks and challenges emerge.

The introduction of virtual practice also raises unique equity concerns, potentially expanding access but in unequal ways [60]. Regulators have a role in ensuring that regulatory decisions do not perpetuate systemic inequities [61]. Our previous research on for-profit virtual care in Canada found that equity has not been adequately considered within standards from medical regulators [6, 22]. Equity considerations must be embedded in regulatory decision-making as technology continues to evolve, and many of these considerations apply to regulatory policies and practices (e.g., virtual discipline hearings) as well as regulating virtual practice.

Our participants also described varied approaches to virtual training experiences and entry-to-practice exams, and this was an area where participants noted disparate approaches were common even across regulators in the same field. A growing body of literature across various professions suggests that there are advantages and lessons to be learned from virtual training models, including virtual reality. More evidence is needed to support informed regulatory decision-making in this evolving area of technology-enabled training and assessing entry-to-practice competency. For nursing, social work, and law, training usually requires hands-on, practical experience that may be difficult to gain in a virtual or simulated setting; however, there may be advantages to innovative virtual training models [62]. There may also be lessons that can be learned from professions, such as paramedicine, that use training via virtual reality to help establish competence [63–65].

### Varied approaches to ensuring technologically competent professionals

As regulators work to strike a balanced approach for enabling professionals to deliver virtual services, practitioners themselves must build technological competence. As discussed by our participants, regulators expect registrants to have the required knowledge and skills related to technology but the approaches regulators have taken to ensure that registrants have this competence differed. Scholars have explored the knowledge needs and competencies that members of their professions require to function in an increasingly digitalized world [59, 66, 67]. As discussed by our participants, technological competence will look different depending on each practice setting. However, with growing interprofessional collaboration, regulators within and across sectors have an opportunity to work together to share evidence and explore how competencies can be harmonized to ensure greater consistency amongst professionals.

Aside from being competent in using technology to deliver professional services, social media introduces implications for how regulated professionals interact with the public to maintain public trust. Many professional regulators have developed standards of practice to outline responsibilities and accountabilities due to increased complaints and findings of unprofessional conduct centred on inappropriate social media use [9, 68–71]. High-profile conduct cases have sparked considerable debate about the need to balance professionals’ freedom of expression and speech with their obligations to maintain public trust. Social media can be a powerful platform for fostering professional networks, continuing education, and advocacy to encourage system improvements. At the same time, it can potentially cause significant social harm. Regulators will continue to need to evaluate how they approach registrants who use social media in ways that push the boundaries of conventional views of professionalism.

### Opportunities to leverage regulatory data

Participants discussed the opportunities that technology can bring in facilitating better use of regulatory data to inform decision-making, though many challenges–including adequate knowledge and resources, addressing privacy concerns, and ensuring data platform interoperability–remain to realize this potential. Beyond the use of data analytics to inform internal regulatory policies and processes, regulators have an opportunity to leverage data to inform broader social goals such as workforce planning. Within the health and social care sectors, the importance of standardized health workforce data for integrated workforce planning has been recognized by scholars [72–74] and has become a priority for federal, provincial, and territorial governments in Canada [75–77]. Promising practices exist in other jurisdictions such as the United States and Australia where national databases and datasets are used, and Canada has recently announced pan-Canadian databases for physician and nursing data [75, 78]. These data support inter-jurisdictional labour mobility and awareness of registration status and malpractice or disciplinary histories. The collection of standardized data can also provide meaningful input on issues such as the mental health of the workforce, a priority mentioned by all our participants. In addition, equity must be a priority, and the collection of enhanced demographic data could facilitate better understanding of the diversity of the workforce and the inequities that exist to inform policy and research needs.

## Limitations and future research

The transferability of these findings should be interpreted carefully when applying to other regulatory contexts within and outside of Canada. Our participant sample was small and meant to provide foundational insights into how regulators are navigating an increasingly digitalized world. Members of our team are currently engaged in two funded research projects that build on this foundational work, one that expands the current study to survey and interview regulators across Canada, and one examining whether ethical codes and guidelines from regulators provide sufficient guidance for practitioners when they encounter challenges related to technology in practice. The implications of technology on professional regulation will require monitoring and evaluation over time to examine how technological innovations impact all facets of regulation and professional work.

## Conclusion

Our case study explored how three Ontario professional regulators operating within different legislative frameworks are navigating technology in the digital era. Cross-sectoral participants provided theoretical and pragmatic insights for regulating in the digital era and the role regulators can and should play in digitally enabled professional practice. These insights provided an understanding of how these regulatory bodies are responding to the challenges of upholding their mandate to regulate in the public interest during rapid digital evolution. Our analysis identified five themes: balancing efficiency potential with risks of certain technological advances; the potential for improving regulation through data analytics; considering how to regulate a technologically competent workforce; recalibrating pandemic emergency measures involving technology; and contemplating the future of technology on regulatory policy and practice. Regulators will continue to grapple with considerations about technology used to facilitate regulatory functions, balancing consumer demands amid emerging risks posed by technology, producing technologically competent workforces, and informing workforce planning via regulatory data. Policymakers and regulators across Canada and internationally should prioritize risk-balanced policies, guidelines, and practice standards for modern professional regulation.

## Supporting information

S1 File Description of regulatory documents. (PDF)

S2 File Semi-structured interview guide. (PDF)

S3 File COREQ checklist. Consolidated criteria for reporting qualitative studies (COREQ): 32-item checklist.
(PDF)
