# Supplementary material for: Professional regulation in the digital era: A qualitative case study of three professions in Ontario, Canada
Source: PLoS One. 2024 May 10;19(5):e0303192. doi: 10.1371/journal.pone.0303192 (PMC11086820; doi:10.1371/journal.pone.0303192)
Supplement: S1 File — (PDF) [file pone.0303192.s001.pdf]

Supplementary File – Documents Included

1. College of Nurses of Ontario (CNO)

Title	Date*	Category	Description	URL**
International Nurse Regulator Collaborative (INRC) Position Statement on Practice Location and Inter-Country Nursing Practice	2015	Position statement	The CNO posted the INRC's position statement on nurses' practicing across geographic borders, including via virtual care. Note: The INRC is a consortium of the following nursing regulators: CNO, the Nursing Council of New Zealand, the Singapore Nursing Board, the Nursing & Midwifery Board of Australia, the College of Registered Nurses of British Columbia, the Nursing & Midwifery Board of Ireland, and the National Council of State Boards of Nursing.	https://www.cno.org/globalassets/docs/prac/inrc-practice-location-postion-statement.pdf
Entry-to-Practice Competencies for registered nurses	2018	Competencies	Includes competencies around social media, information and communication technologies, nursing informatics, and emerging technologies.	https://www.cno.org/globalassets/docs/reg/41037-entry-to-practice-competencies-2020.pdf
Entry-to-Practice Competencies for Registered Practical Nurses	2019	Competencies	Includes competencies around information and con technologies and social media.	https://www.cno.org/globalassets/docs/reg/41042_entrypracrpn-2020.pdf
Practice Guideline: Telepractice	2020	Practice guidance	Outlines nurse accountabilities to guide virtual practice.	https://www.cno.org/globalassets/docs/prac/41041_telephone.pdf
Briefing for the Dec 3, 2020 Council meeting	2020	Council Material	Includes discussion notes about the effort to modernize practice standards and keep pace with changes.	https://www.cno.org/globalassets/1-whatiscno/council/meetings/2020/meeting-materials-observer-package.pdf
Modernizing Standards	2020-2022	Practice standards	Webpage about ongoing work to modernize all practice standards. This work led to the new CNO code of conduct (effective in 2023).	https://www.cno.org/en/trending-topics/modernizing-standards/

Title	Date*	Category	Description	URL**
Council Minutes	2021 (Dec 2)	Council Material	Includes minutes of discussion on modernizing practice standards effort.	https://www.cno.org/globalassets/1-whatiscno/council/meetings/2021/council-minutes-202112.pdf
Strategic Plan 2021-2024	2021	Innovation	In the Strategic Plan, one of CNO's four pillars is building and operating an Insights Engine focusing on data, analytics, and insights.	https://www.cno.org/globalassets/docs/general/strategic-plan-2021.pdf
Code of Conduct	2022 (Dec 7)	Practice standards	New Code of Conduct. Posted in 2022 but officially came into effect in June 2023.	https://www.cno.org/globalassets/docs/prac/49040_coc_effective_202306.pdf

2. Law Society of Ontario (LSO)

Title	Date*	Category	Description	URL**
Frequently Asked Questions about Client Identification and Verification	2020-2022 (version collected was last updated Jan 22, 2022)	Practice guidance	Update to registrants on rules around virtual verification of identity (e.g., during the COVID-19 emergency).	https://lso.ca/paralegals/practice-supports-and-resources/topics/the-paralegal-client-relationship/identification-and-verification/appendix-8%c2%a0%c2%a0%c2%a0%c2%a0questions-and-answers
Law Society implements online delivery of licensing examinations	2020 (May 5)	News/media release	Update on online delivery of licensing exams during COVID-19 emergency.	https://lso.ca/gazette/news/law-society-implements-online-delivery-of-licensin
Practice management guideline: Technology	2020 (Jul 31)	Practice guidance	Guidance on technology use in practice, including security issues, disaster management, and technological obsolescence.	https://lso.ca/lawyers/practice-supports-and-resources/practice-management-guidelines/technology

Title	Date*	Category	Description	URL**
Technology Task Force	2021	Innovation	Technology task force webpage with links to resources and reports.	https://lso.ca/about-lso/initiatives/technology-task-force
Remote commissioning	2021 (Jan 8)	Practice guidance	Rules regarding remote or virtual commissioning of documents, including links to supporting resources and best practices.	https://lso.ca/lawyers/practice-supports-and-resources/topics/the-lawyer-client-relationship/commissioner-for-taking-affidavits-and-notary-publ/l%e2%80%99attestation-a-distance
Technology Task Force: Report on Regulatory Sandbox for Innovative Technological Legal Services	2021 (Apr 22)	Innovation	LSO's Technology Task Force recommended the creation of a regulatory sandbox for innovative technological legal services ("ITLS") as a five-year pilot.	https://lawsocietyontario.azureedge.net/media/lso/media/about/convocation/2021/convocation-april-2021-technology-task-force-report.pdf
By-Law 16	2021 (Apr 22)	Innovation	New LSO by-law to allow participation in the regulatory sandbox.	https://lso.ca/about-lso/legislation-rules/by-laws/by-law-16
Renewing the Law Society's Continuing Competence Framework	2021 (June)	Competencies	Update and report on continuing competence framework, including discussion of technological competence as one of the key themes that may inform new approaches to competence.	https://lawsocietyontario.azureedge.net/media/lso/media/about/convocation/2021/convocation-june-2021-competence-taskforce-report.pdf
A2I pilot project website	2021 (Oct)	Innovation	Access to Innovation (A2I) is a five-year pilot project which allows providers of innovative technological legal services to serve consumers while complying with operating conditions that protect the public.	https://lso.ca/about-lso/access-to-innovation
Law Society's Access to Innovation project is accepting applications	2021 (Nov 3)	News / Media releases	News release about the A2I program accepting applications.	https://lso.ca/news-events/news/latest-news-2021/law-society%E2%80%99s-access-to-innovation-project-is-acce

**Title**	**Date***	**Category**	**Description**	**URL****
Key outcomes: Access to Justice Week	2021 (Nov 4)	News / Media release	Update on A2I project and links to news releases.	https://lawsocietyontario.azureedge.net/media/lso/media/lso-media-room/documents/a2j-week-2021-outcomes_final_en.pdf
Investigation into cheating on the Law Society's licensing examinations	2022	Entry to practice	Update on investigation into cheating that occurred on the LSO's virtual licensing exam.	https://lso.ca/news-events/news/latest-news-2022/decisions-rendered-in-investigation-into-cheating
Renewing the Law Society's Continuing Competence Framework	2022 (May)	Competencies	Link to the approved new Competency Framework that includes creating a mandatory online practice essentials course for sole practitioners and adopting the commentary to the rules of professional conduct regarding technological competence.	https://lso.ca/about-lso/initiatives/competence-framework#:~:text=Convocation%20approved%20the%20Final%20Report,practitioner%20for%20the%20first%20time
COVID emergency measure (virtual verification) extended to Jan 1, 2024	2022 (Nov 22)	News/media releases	Update on COVID-19 emergency measures regarding virtual identity verification.	https://lso.ca/news-events/news/latest-news-2022/covid-emergency-measure-(virtual-verification)-ext
Technology Resource Centre	N/A	Practice guidance	Webpage with all LSO practice supports, training, and CPD programming focused on technology.	https://lso.ca/lawyers/technology-resource-centre

3. Ontario College of Social Workers and Social Service Workers (OCSW)

Title	Date*	Category	Description	URL**
If providing services by electronic means, what platform does the college recommend?	N/A	Practice guidance	The OCSW does not recommend a specific platform, provides links to other OCSW resources about practicing virtually.	https://www.ocswssw.org/ocswssw-faq/if-providing-services-by-electronic-means-what-platform-does-the-college-recommend/
Communication Technology Practices and Policies for a Digital World	2017 (Fall)	Practice guidance	Article in the OCSW newsletter that interprets the standards of practice for OCSW registrants considering technology-enabled practice.	https://www.ocswssw.org/wp-content/uploads/PN-Communication_Technology_Practices_Policies_for_Digital_World.pdf
Information & Communication Technology in Social Work Practice	2019 (Jun 23)	Practice guidance	Presentation slides on technology in social work practice for OCSW registrants.	https://www.ocswssw.org/wp-content/uploads/Information_Communication_Technology.pdf
Top 10 Considerations for Using Communication Technology in Practice	2019 (Fall)	Practice guidance	Article in OCSW newsletter that provides top 10 considerations for using communication technology in social work practice.	https://www.ocswssw.org/wp-content/uploads/OCSWSSW_Perspective_Fall_2019-EN.pdf
Strategic Plan 2020-2023	2020-2023	Innovation	One of OCSW's priorities is to enhance regulatory efficiency by leveraging technology and making registration processes available online. The strategic plan also includes how the regulator will know it has achieved its vision, including that "there is a fully integrated technological platform	https://www.ocswssw.org/wp-content/uploads/Strategic_Plan_2020-2023_EN.pdf

Title	Date*	Category	Description	URL**
			to increase transparency of College business and processes to promote public protection" (p. 6).	
Top 6 Considerations for Virtual Services	2020 (Fall)	Practice guidance	Article in OCSW newsletter that provides top 10 considerations for providing virtual services.	https://www.ocswssw.org/2020/11/19/top-6-considerations-for-virtual-services/
2021 Annual report	2021	Innovation	Description of changes made to enhance regulatory effectiveness using technology, including online registrant portal and website redesign.	https://www.ocswssw.org/wp-content/uploads/2021-Annual-Report-EN.pdf
The Evolving Landscape of Electronic Practice	2021 (Fall)	Practice guidance	Article in OCSW newsletter based on professional and ethical obligations for innovative social work practice using technology.	https://www.ocswssw.org/2021/11/21/practice-notes-the-evolving-landscape-of-electronic-practice/
Professional Practice Update: Increasing Accessibility and Equity for Clients	2021 (May 10)	Practice guidance	Report on a survey of OCSW registrants that noted a dramatic shift from in-person to virtual services and highlighted concerns around accessibility and equity arising from gaps in clients' access to technology. Provides link to the Ontario Association of Social Workers' webpage on programs and services to support access to technology-enabled social work practice.	https://www.ocswssw.org/2021/05/10/professional-practice-update-increasing-accessibility-and-equity-for-clients/
Practicing Electronically in Ontario	N/A	Entry to practice	Resources for social workers outside of Ontario who wish to practice in Ontario by electronic means. OCSW offers a license limited to electronic practice.	https://www.ocswssw.org/applicants/online-application/practising-electronically-in-ontario/

*Date for documents is based on what was accessed during data collection (initially conducted May – September 2022 and updated in January 2023 before interviews)
**URLs were valid at the time of data collection. Please contact the first author at kleslie@athabascau.ca for any documents no longer accessible at the linked URL.
