# Supplementary material for: Professional regulation in the digital era: A qualitative case study of three professions in Ontario, Canada
Source: PLoS One. 2024 May 10;19(5):e0303192. doi: 10.1371/journal.pone.0303192 (PMC11086820; doi:10.1371/journal.pone.0303192)
Supplement: S2 File — (PDF) [file pone.0303192.s002.pdf]

S1. Semi-Structured Interview Guide

Potential Discussion Categories	Guiding Questions	Potential Follow-Up Questions/Prompts
**Entry to practice**	What challenges has your regulatory body experienced related to entry to practice with increasing digital options for education/licensing exams?<br><br>How can entry-to-practice competencies adequately address technologically enabled practice for the profession you regulate?	Has there been an evaluation of new registrants' preparedness, performance, patient outcomes and complaints?<br><br>Have any adjustments to curriculum/training been made or considered to prepare for digitally enabled practice environments or service delivery?
**Standards of practice/ technological competencies**	Has the increase in technology-enabled practice changed other aspects of practice standards?<br><br>Is technological competence addressed in requirements for meeting the standards of practice?	What are the potential implications of the digital era for the client/provider relationship?<br><br>Has your regulatory body considered how registrants should be accountable for the use of technology in practice (e.g., if relying on AI systems that are using biased or inaccurate information)?
**Continuing Competence**	Have changes to continuing competence requirements been made to include technology-enabled practice?	Did continuing competence requirements change during the pandemic?
**Virtual practice**	Noting areas where regulators have made changes to policy and practice in regulating virtual service provision, can you speak to which areas were the greatest priority and why?<br><br>How are cross-jurisdictional practice/licensure or mobility issues being addressed?	Are there any specific areas of focus (e.g., risk, equity, access, competence) for you as a regulator when considering how to regulate virtual practice? What is next in terms of regulating virtual practice?
**Social media**	How does your regulatory body approach the social media use of registrants?	How does your regulatory body approach the use of social media by the regulator itself?
**Legislation and policy issues**	Does governing legislation provide sufficient flexibility for regulators to respond quickly to emerging risks?	Has flexibility or nimbleness in the legislative framework taken on a different importance with the increasing pace of change related to technology?

	Could you speak to how the legislative framework or any particular structures/processes enabled the regulation of virtual practice?	
**Discipline**	How has technology impacted investigations and discipline hearings?	Are any temporary measures implemented during the pandemic (e.g., online/virtual hearings) being discussed within the regulator as important to retain?
**Identifying risks**	In times of increased workforce challenges and concerns about the mental health of the workforce, how does the regulator think about risks to the public/the public interest?<br><br>How does the regulator identify emerging risks to the public around technology? What role does technology play in helping regulators identify emerging risks?	What is the regulator's role in addressing inequities?
**Regulatory innovation and use of data**	Can you identify the regulator's most successful innovation related to technology-enabled practice?<br><br>What role does technology or data analytics play in improving regulatory processes?	
**Collaboration with other regulators**	Have collaborations nationally and internationally been facilitated with technology?<br><br>How do learnings from other jurisdictions or professions influence your work?	Were these collaborations accelerated through the pandemic?<br><br>Can you describe the extent to which you are collaborating on various initiatives?
**Concluding thoughts**	Is there anything else you would like us to know about how your regulatory body has been regulating in the digital era?	
